# Optimizing maternal and neonatal outcomes through tight control management of inflammatory bowel disease during pregnancy: a pilot feasibility study

**DOI:** 10.1038/s41598-023-35332-z

**Published:** 2023-05-22

**Authors:** Rohit Jogendran, Katie O’Connor, Ajani Jeyakumar, Parul Tandon, Geoffrey C. Nguyen, Cynthia Maxwell, Vivian Huang

**Affiliations:** 1 Division of Gastroenterology and Hepatology, IBD Clinical Research Program, Mount Sinai Hospital, Sinai Health System, University of Toronto, Suite 441 - 600 University Avenue, Toronto, ON M5G 1X5 Canada; 2 Division of Maternal Fetal Medicine, Department of Obstetrics & Gynaecology, Mount Sinai Hospital, Toronto, ON Canada; 3 Women’s College Hospital Research Institute, Women’s College Hospital, Toronto, ON Canada

**Keywords:** Gastroenterology, Inflammatory bowel disease

## Abstract

A home point-of care FCP test (IBDoc) and a self-reported clinical disease activity program (IBD Dashboard) may improve routine monitoring of IBD activity during pregnancy. We aimed to evaluate the feasibility of tight control management using remote monitoring in pregnant patients with IBD. Pregnant patients (< 20 weeks) with IBD were prospectively enrolled from Mount Sinai Hospital between 2019 and 2020. Patients completed the IBDoc and IBD Dashboard at three core time points. Disease activity was measured clinically using the Harvey–Bradshaw Index (mHBI) for CD and partial Mayo (pMayo) for UC, or objectively using FCP. A feasibility questionnaire was completed in the third trimester. Seventy-seven percent of patients (24 of 31) completed the IBDoc and IBD Dashboard at all core time points. Twenty-four patients completed the feasibility questionnaires. All survey respondents strongly preferred using the IBDoc over standard lab-based testing and would use the home kit in the future. Exploratory analysis identified discordance rates of more than 50% between clinical and objective disease activity. Tight control management using remote monitoring may be feasible among pregnant patients with IBD. A combination of both clinical scores and objective disease markers may better predict disease activity.

## Introduction

Inflammatory bowel disease (IBD) is a chronic intestinal inflammatory condition that affects people of reproductive age^1,2^. Managing IBD during pregnancy is challenging, as pregnancy-related gastrointestinal symptoms can often overlap with symptoms of active IBD. Unfortunately, active disease can have serious adverse effects on pregnancy outcomes and as such, there remains a critical need to assess and manage disease activity during this high-risk period^3,4^.

Current clinical guidelines recommend optimizing IBD disease activity to minimize the risk of disease relapse and pregnancy-related outcomes^5,6^. Although endoscopy remains the standard of practice for assessing IBD disease activity and can be performed in pregnant people if indicated^7–9^, non-invasive tests are generally preferred to avoid procedure-related risks. As a result, objective biomarkers of inflammation have emerged as a promising alternative^10,11^. Fecal calprotectin (FCP) is a non-invasive inflammatory marker and strong predictor of disease relapse, histological remission, and mucosal healing^12–18^.

Emerging studies have now confirmed that tight control management using a combination of both clinical symptoms and objective biomarkers leads to improved clinical and endoscopic outcomes^19^. However, there has been no such trial in pregnant patients with IBD where control of disease activity is strongly recommended^5,6^. It has been previously established that close monitoring of clinical disease activity in pregnant people with IBD results in improved clinical outcomes^20^. Recent studies have demonstrated that pregnancy itself does not alter FCP levels and that FCP correlates with IBD disease activity^21–24^. However, regular assessments for monitoring and controlling objective IBD disease activity can be impractical for many pregnant patients with IBD due to limited resources and challenges with access to care. In particular, health insurance plans may not provide coverage for lab-based FCP testing, and require patients to return stool samples via mail or in-person**.**

Patient eHealth programs have emerged as an attractive platform to complement routine clinical care in the management of chronic diseases. Recent studies have demonstrated the effective use of eHealth strategies in improving medication adherence and reducing health care utilization in patients with IBD^25–27^. IBDoc, a home point-of-care rapid lateral assay FCP test can be used to monitor objective IBD disease activity in a timelier fashion, without requiring patients to return stool samples^28^. Previous studies on IBDoc have demonstrated that home point-of-care testing is feasible and acceptable to patients^29–31^, however, it is unknown whether tight control management using an eHealth program is beneficial for routine monitoring of pregnant patients with IBD. The aim of our study was to evaluate feasibility of tight control management using remote monitoring in IBD during pregnancy.

## Methods

This was a prospective pilot study performed at the Mount Sinai Hospital (MSH), an academic, tertiary care center. It was approved by the institutional review and health research ethics board at MSH. All methods were performed in accordance with relevant guidelines and regulations.

### Study participants

Pregnant patients, aged ≥ 18 years of age, with IBD (Crohn’s Disease (CD) or ulcerative colitis (UC)) were recruited from the Preconception and Pregnancy in IBD clinic at MSH. Patients at less than 20 weeks of gestation with a singleton pregnancy were identified and included. Patients were required to have access to a smart phone and internet for the IBDoc FCP program and IBD Dashboard for self-reported symptoms. Exclusion criteria included patients who had any prior IBD surgery or symptomatic strictures or perianal disease, medication changes within eight weeks of the screening visit or on a steroid taper, and the inability to speak and understand English. Patients completed the home point-of-care FCP testing and self-reported IBD-related symptoms at three core time points, (1) screening/baseline at either the first or second trimester (< 20 weeks), (2) second trimester (14–27 weeks) if a baseline was completed in first trimester, and (3) third trimester (28–32 weeks). All subjects were emailed and/or called by the research coordinator at least 3 times with reminders for each study time point. Subjects that did not adhere to the study visits were asked (if they were reachable) why they did not complete study activities.

### Variables and data measurement

At the screening visit, the following data was recorded: (a) patient demographics including age, relationship status, educational status, socioeconomic status, reproductive history, and social habits, (b) IBD characteristics such as diagnosis, phenotype (Montreal Classification), past and current medication exposure (5-aminosalicylates (5-ASA), corticosteroids, thiopurines (6-mercaptopurine and azathioprine), anti-tumor necrosis factor (TNF), vedolizumab, or ustekinumab), (c) past medical history including medications and surgeries, and (d) access to technology. The modified HBI (Harvey Bradshaw Index)^32^ for CD and partial Mayo Index (pMayo)^33^ for UC were used for clinical assessment of disease activity, while FCP was measured for objective assessment of disease activity. At the initial visit, patients were provided with an IBDoc kit and instructions on completing a baseline FCP at home.

### Tight control management

**Figure 1:**
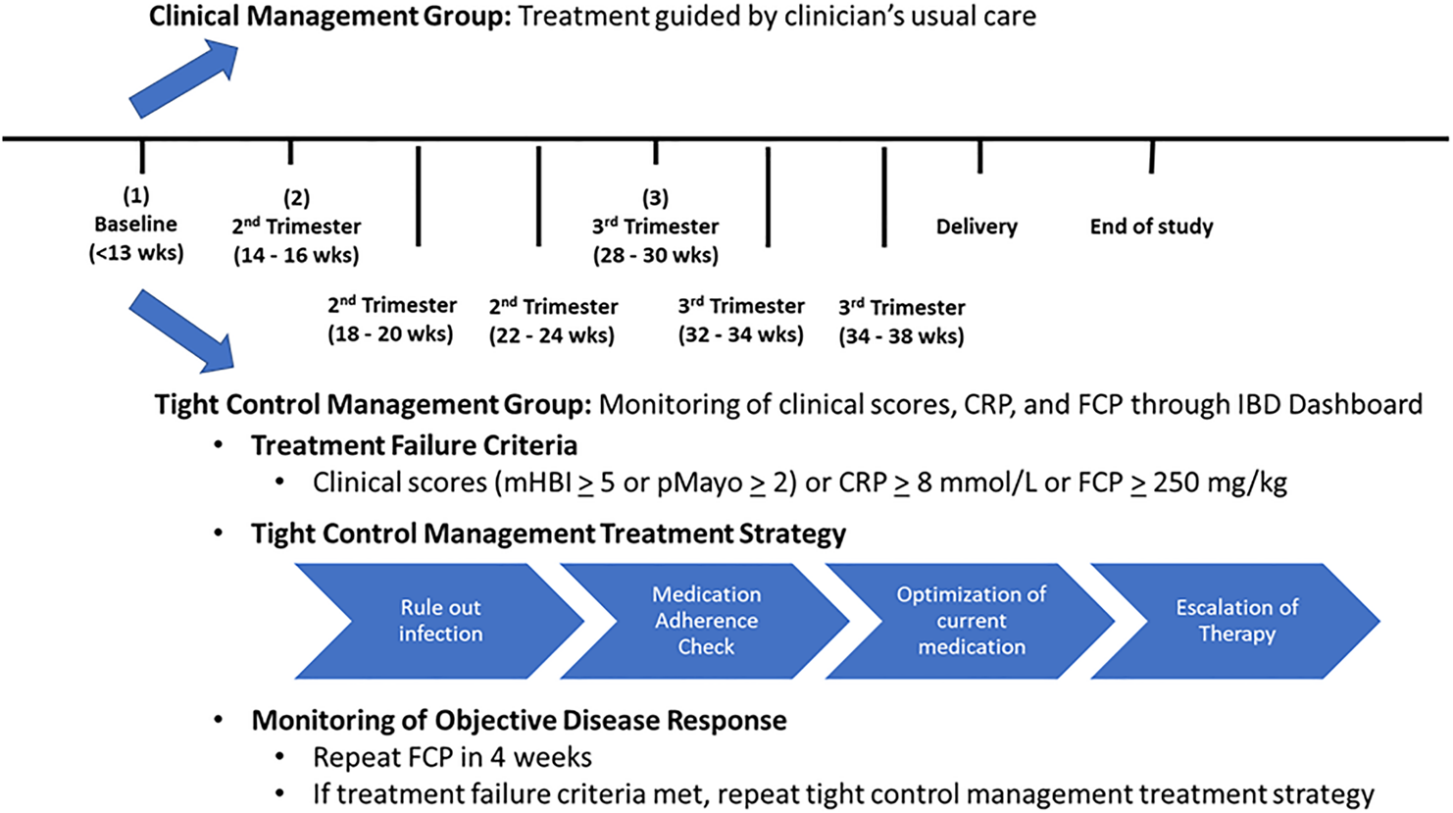
Algorithm for tight control management treatment strategy.

Tight control management was the protocolized remote monitoring of both clinical and objective IBD disease activity with subsequent management of active disease. At each time point, disease activity was monitored clinically and objectively for evidence of treatment failure. The criteria for treatment failure included either (1) active clinical disease defined as a mHBI ≥ 5 or pMayo ≥ 2, or (2) active objective disease defined as a FCP ≥ 250 µg/g. Treatment failure during this period triggered a tight control management treatment strategy, which included diagnostic investigations (i.e., rule out infections), medication adherence (optimization of current medication dose or frequency), or escalation of therapy. Any changes in therapy or diagnostic investigations were at the discretion of the clinical team. The IBDoc FCP and IBD Dashboard were repeated in four weeks to monitor for objective and/or clinical disease response to treatment. If the repeated clinical scores normalized or IBDoc FCP was < 250 µg/g, the patient continued their current management to the next study time point. However, if the repeated clinical scores remain elevated or IBDoc FCP was ≥ 250 µg/g, the tight control management treatment strategy was cycled again until scores normalized or to the next time point (Fig. 1).

### User feasibility patient questionnaire

At the third trimester, patients were asked to complete a usability questionnaire (Appendix 1). A 5-point Likert scale (1 = “strongly disagree”, 5 = strongly agree) was used to score 7 statements in the IBDoc questionnaire and 13 statements in the IBD Dashboard questionnaire.

### Buhlmann IBDoc FCP and IBD dashboard

The Buhlmann IBDoc is an in vitro diagnostic immunoassay for home point-of-care FCP testing (www.ibdoc.net). A stool sample from the first bowel movement of the day was extracted using the Calex Valve and loaded onto the IBDoc test cassette. The IBDoc test cassette was analyzed by a secure, downloadable smartphone application, CalApp, which was linked to the IBDoc Portal for direct monitoring by health care providers. The assay results were displayed on the smartphone application as a quantitative measurement (only for FCP values between 30 and 1000 µg/g) and categorized into three levels: normal < 100 µg/g (green), 100–250 µg/g (yellow), and > 250 µg/g (red).

Patients were also enrolled into the IBD Dashboard program which allowed for tight control monitoring. The IBD Dashboard is a secure web-based portal for patients to submit their clinical disease activity scores, quality of life, and medication adherence reports in each trimester of pregnancy for review by health care providers^34^. The IBD dashboard was also designed to extract results from the IBDoc portal, and thus contained FCP results from multiple testing points. Furthermore, CRP values (if done) from routine bloodwork were entered into the IBD dashboard for review. The IBD Dashboard informed the research term when patients triggered the criteria for treatment failure.

### Outcomes

The primary outcome of this study was to evaluate feasibility of tight control management via remote monitoring through (1) the proportion of users who completed all core study time points including the IBDoc FCP test and IBD dashboard clinical symptoms and (2) the proportion of users with active disease who completed additional tight control monitoring time points. The secondary outcome was to assess the usability and satisfaction of a remote monitoring program through patient questionnaires.

Clinical outcomes assessed included discordance between objective markers of inflammation and self-reported clinical symptoms. Maternal outcomes recorded included delivery method (vaginal or cesarean), postpartum infection, IBD flare, and hospitalizations post-delivery. Neonatal outcomes assessed included preterm birth (defined as birth before 37 weeks), and low birth weight (LBW, birth weight < 2500 g).

### Statistical analysis

All statistical analysis was performed using the SPSS software version 26 (Chicago, IL). Statistical difference between groups was tested using the student T-test for parametric variables and Mann–Whitney-U test for non-parametric variables. Categorical variables were presented as proportions and assessed for statistical difference via a Pearson chi-square test. Median values with interquartile ranges (IQR) and mean values with standard deviations (SD) were calculated for all continuous variables. A two-sided *p *value < 0.05 was defined as statistically significant. We a priori propose that the tight control monitoring protocol will be considered feasible if more than 75% of subjects complete the core study time points, and more than 75% of those who have active disease complete the additional tight monitoring time points. The second level of feasibility will be 90% of participants completing 75% of the time points. For this pilot study, we estimate that with 20 to 30 subjects, we will be able to obtain feedback regarding patient acceptance of the home stool test monitoring, and adherence. We aimed to enroll 30, which has generally been accepted for feasibility studies.

### Informed consent

Informed consent was obtained from all individual participants included in the study.

## Results

### Baseline characteristics

**Figure 2:**
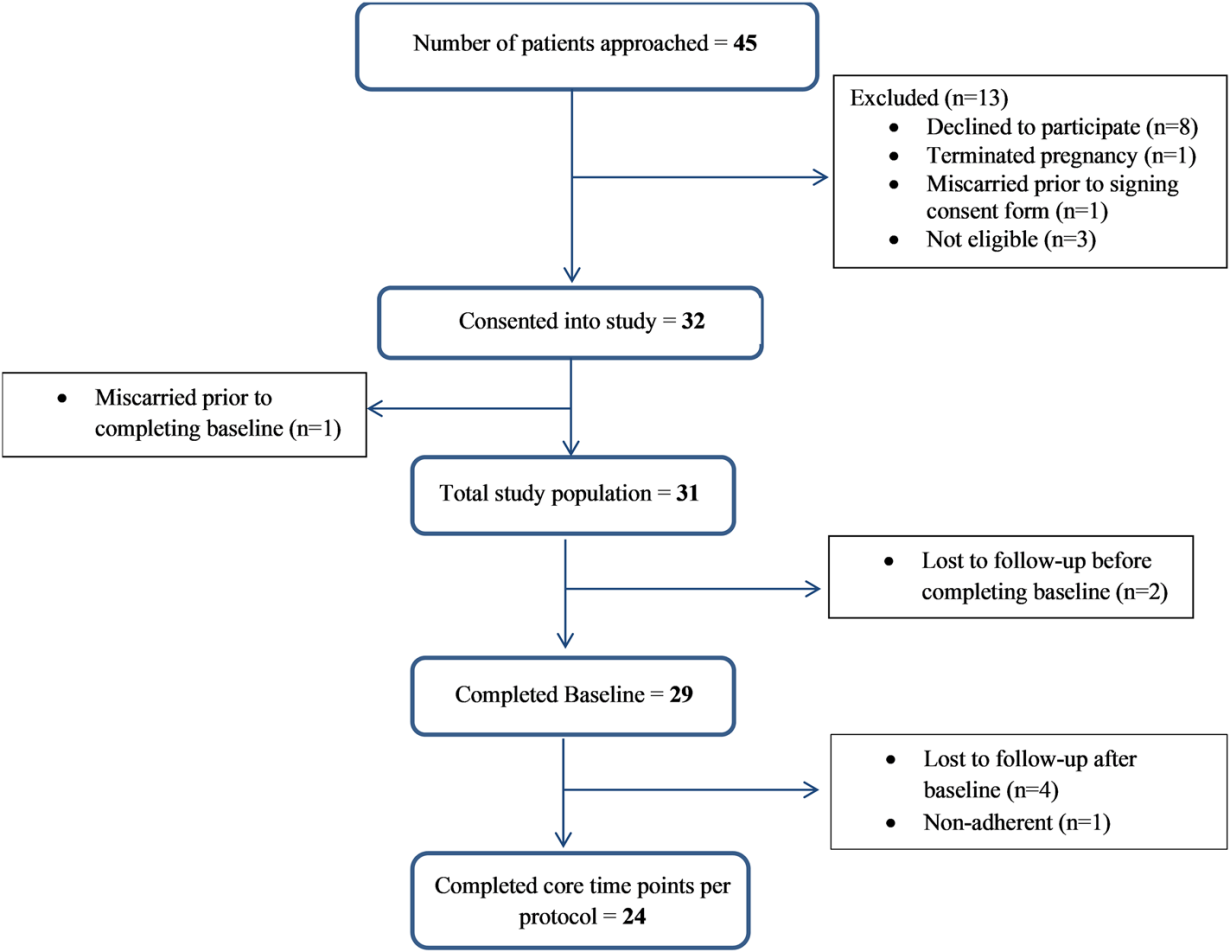
Flow diagram of study participants.

A total of 45 pregnant patients with IBD were approached between September 2019 and October 2020 and 32 consented to participate in this study (Fig. 2). Eight of the 13 excluded patients declined to participate, three did not meet the inclusion criteria, one miscarried prior to consenting, and one had a termination of pregnancy. Of the 32 consented patients, two were lost to follow-up before the baseline time point and one miscarried prior to completing a baseline, leaving a total of 29 patients (17 CD, 12 UC) who consented and completed a baseline test. These were the subjects included in further analyses. The median age at IBD diagnosis was 22.50 (IQR 19.75–27.25) and the median gestational age at time of enrollment was 11.5 weeks (IQR 8.0–18.0, range: 4–20 weeks). All baseline characteristics are described in Table 1.

**Table 1:** Baseline characteristics of all included patients.

	Number of patients (n, %)
Number of patients	
Inflammatory bowel disease	31 (100.0)
Crohn’s disease	18 (58.1)
Ulcerative colitis	13 (41.9)
Median gestational age at enrollment (IQR)	11.5 (8.0–18.0)
Prior pregnancy	
Yes	17 (54.9)
Primiparous	4 (12.9)
Multiparous	4 (12.9)
No	13 (41.9)
Unknown	1 (3.2)
Ethnicity	
Caucasian	25 (80.6)
Asian (Filipino, Japanese, Korean, Chinese)	1 (3.2)
South-Asian	2 (6.5)
Mixed race	2 (6.5)
Unknown	1 (3.2)
Educational status	
Less than high school	1 (3.2)
Completed high school	1 (3.2)
Completed trade school	2 (6.5)
Completed university	12 (38.7)
Completed postgraduate	14 (45.2)
Unknown	1 (3.2)
Marital status	
Single	2 (6.5)
Married	24 (77.4)
Common law	4 (12.9)
Unknown	1 (3.2)
Income	
$20,000-$39,999	2 (6.5)
$40,000-$69,999	8 (25.8)
$70,000-$99,999	3 (9.7)
$100,000 or more	15 (48.3)
Unknown	3 (9.7)
Disease characteristics	
Medication exposure	
No medications	3 (9.7)
5-aminsalicylates (oral or topical)	12 (38.7)
Thiopurines	2 (6.5)
Oral prednisone	1 (3.2)
Budesonide	1 (3.2)
Biologics	11 (35.5)
Anti-TNF	8 (25.8)
Infliximab	5 (16.1)
Adalimumab	3 (9.7)
Anti-TNF + 5-ASA combination	2 (6.5)
Ustekinumab	2 (6.5)
Vedolizumab	1 (3.2)
Ulcerative colitis	
E1-Proctitis	3 (9.7)
E2-Left-sided disease	3 (9.7)
E3-Extensive colitis	5 (16.1)
Crohn’s disease	
Disease location	
L1-Ileal	5 (16.1)
L2-Colonic	4 (12.9)
L3-Ileocolonic	8 (25.8)
Disease behaviour	
B1-Nonstricturing, nonpenetrating	8 (25.8)
B2-Stricturing	4 (12.9)
B3-Penetrating	2 (6.5)
B2 + B3	2 (6.5)
p-perianal disease modifier	3 (9.7)

### Feasibility and user satisfaction

Twenty-nine patients completed the baseline time point, fourteen (48%) in first trimester and fifteen (52%) in second trimester. Unfortunately, one patient who enrolled in first trimester miscarried before the second trimester core time point. Of the remaining 13 patients who enrolled in first trimester, 12 (92%) completed a second trimester core time point, while only 10 (77%) completed a third trimester core time point (Table 2). However, of the 15 patients who enrolled in second trimester, 13 (87%) completed a third trimester core time point. One patient who enrolled in first trimester delivered prematurely at 29 weeks gestational age. Overall, 24 of 31 (77%) patients were adherent and completed all core time points. We identified that 6 of 7 (86%) patients were lost to follow-up—four were unresponsive, one had a family emergency, and one had an unplanned pregnancy. We determined that 1 of 7 (14%) patients were non-adherent following admission to hospital for hypertensive emergency.

**Table 2:** Adherence of participants to study time points.

Enrollment	Time point (n)*	Completed (n,%)	Missed	Active disease at time point
Trimester 1 (n = 14)	Trimester 1 (n = 14)	14 (100%)	0	7
	Trimester 2 (n = 13)	12 (92%)	1 (Family emergency)	3
	Repeat Time Point 1 (n = 3)	2 (67%)	1 (Lost to follow-up)	0
	Repeat Time Point 2 (n = 0)	–	–	–
	Trimester 3 (n = 13)	10 (77%)	3 (n = 2: Lost to follow-up, n = 1: Non-adherent)	1
	Repeat Time Point 1 (n = 1)	1 (100%)	0	1
	Repeat Time Point 2 (n = 1)	0 (0%)	1 (Patient was worried of a potential high result close to delivery)	–
Trimester 2 (n = 15)	Trimester 2 (n = 15)	15 (100%)	0	6
	Repeat Time Point 1 (n = 6)	4 (67%)	2 (Lost to follow-up)	2
	Repeat Time Point 2 (n = 0)	–	–	–
	Trimester 3 (n = 15)	13 (87%)	2 (Lost to follow-up)	4
	Repeat Time Point 1 (n = 4)	3 (75%)	1 (Repeat time point coincided with delivery)	2
	Repeat Time Point 2 (n = 2)	1 (50%)	1 (Patient was worried of a potential high result close to delivery)	1

Active disease: Elevated FCP (fecal calprotectin) ≥ 250 μg/g, mHBI ≥ 5 or pMayo ≥ 2.

*n = number expected to complete each time point; non-adherent = patient did not complete time point despite reminders from research team.

We compared the proportion of patients with active disease who completed the additional tight control monitoring time points. At the second trimester core time point, 9 of 29 (31%) patients had evidence of active disease (Table 2**)**. Of these nine patients, 6 (67%) completed the first additional monitoring time point in second trimester. Two patients continued to have active disease at this time point, however, since the second additional monitoring time point in second trimester coincided with third trimester, these patients proceeded to a third trimester core time point. Roughly seventeen percent of patients (5/29) had evidence of active disease at the third trimester core time point. Four of five (80%) patients completed the first additional monitoring time point in third trimester, however, three patients continued to have active disease. Only one-third of these patients completed the second additional monitoring time point in third trimester. Overall, the total proportion of additional monitoring time points completed amongst study participants was 65% (11/17). We further determined that 11 of 29 (38%) patients triggered the protocol for additional tight control monitoring time points, and that only 6 of 11 (55%) patients were adherent. Of these five non-adherent patients, two were anxious of having a high FCP result near delivery, two were lost to follow-up from the core time points, and one had their additional time point coincide with delivery.

We then evaluated the usability and user satisfaction of a remote monitoring program for pregnant patients with IBD. Twenty-four patients completed the IBDoc and IBD Dashboard questionnaires (77% response rate). More than 95% of participants either strongly agreed or agreed that the IBDoc and IBD Dashboard were easy to use (Table 3**)**. In addition, 23 (96%) and 19 (79%) of participants either strongly agreed or agreed that the IBD Dashboard and the IBDoc were user friendly, respectively. Most importantly, all survey respondents strongly agreed that they preferred using the home point-of-care IBDoc stool kit over standard lab-based tests, would use the tool kit in the future, and would recommend to other patients. Similarly, 21 (88%), 22 (92%), and 22 (92%) of participants either strongly agreed or agreed that they enjoyed using the IBD Dashboard, would use in the future, and would recommend to other patients, respectively (Table 3**).**

**Table 3:** IBDoc and IBD Dashboard feasibility questionnaire results.

	Median (IQR)
IBDoc questions	
1. The IBDoc tool kit is easy to use	5.0 (1.0)
2. The IBDoc tool kit makes completing stool tests convenient	5.0 (0.0)
3. I could easily fit the time it took to complete the IBDoc test into my schedule	5.0 (1.0)
4. The IBDoc App is user friendly	5.0 (1.0)
5. I prefer completing at home stool tests using the IBDoc tool kit (compared to taking samples to lab or clinic)	5.0 (0.0)
6. I would use the IBDoc tool kit in the future	5.0 (0.0)
7. I would recommend the IBDoc tool kit to other patients	5.0 (0.0)
IBD dashboard questions	
1. The IBD Dashboard is easy to use	5.0 (1.0)
2. The IBD Dashboard is user friendly	5.0 (0.5)
3. The IBD Dashboard had too many questions	1.0 (1.0)
4. The questions on the IBD Dashboard took an appropriate amount of time to complete	5.0 (0.0)
5. I could easily report my clinical symptoms and medication intake on the IBD Dashboard	5.0 (1.5)
6. I could easily fit the time it took to complete the tasks on the IBD Dashboard into my schedule	5.0 (0.0)
7. The IBD Dashboard is helpful for tracking my symptoms	4.0 (2.0)
8. I feel comfortable providing my clinical symptoms and medication intake on the IBD Dashboard	5.0 (0.5)
9. The email reminders from the IBD Dashboard are useful	5.0 (1.0)
10. The IBD Dashboard is useful for monitoring disease	5.0 (1.25)
11. I liked using the IBD Dashboard	5.0 (1.0)
12. I would use the IBD Dashboard in the future	5.0 (1.0)
13. I would recommend the IBD Dashboard to other patients	5.0 (1.0)

### Characteristics of patient non-adherence

We explored feasibility and patient factors amongst those who were non-adherent to core and additional monitoring time points. A total of 50% of non-adherent patients (4/8) completed a feasibility questionnaire, and all survey respondents strongly agreed that they preferred using the home point-of-care IBDoc stool kit, would use the tool kit in the future, and would recommend it to other patients. We identified that 50% of non-adherent patients with prior pregnancies (3/6) had previous pregnancy-related complications including miscarriage and tubal pregnancy (Table 4). In addition, 25% of patients (2/8) declined completing additional third trimester monitoring time points due to concerns of a high FCP result near the time of delivery. We also assessed pregnancy-related outcomes of those patients who were non-adherent to the intervention time points. We determined that 12.5% of non-adherent patients (1/8) developed an IBD flare following delivery, and 12.5% (1/8) developed a postpartum infection.

**Table 4:** Characteristics of all non-adherent patients at core and additional time points.

	Number of patients (n, %)
Number of patients	
Inflammatory bowel disease	8 (100.0)
Crohn’s disease	5 (62.5)
Ulcerative colitis	3 (37.5)
Median gestational age at enrollment (IQR)	10.5 (8.5–17.0)
Education	
Completed postgraduate	6 (75.0)
Completed university	1 (12.5)
Less than high school	1 (12.5)
Income	
$100,000 or more	4 (50.0)
$70,000-$99,999	2 (25.0)
$40,000-$69,999	1 (12.5)
Unknown	1 (12.5)
Prior pregnancy characteristics	
Previous pregnancy	
Yes	6 (75.0)
No	2 (25.0)
Pregnancy-related outcomes*	
Live Birth	3 (42.9)
Miscarriage	3 (42.9)
Tubal pregnancy	1 (14.2)
Current pregnancy characteristics	
Delivery Method	
Vaginal induced	4 (50.0)
Unknown	4 (50.0)
Term at delivery	
Full term	4 (50.0)
Unknown	4 (50.0)
Postpartum infection	
Yes	1 (12.5)
No	3 (37.5)
Unknown	4 (50.0)
IBD Flare following delivery	
Yes	1 (12.5)
No	3 (37.5)
Unknown	4 (50.0)

*Total Number of Pregnancies amongst 6 previous pregnant patients was 7.

### Clinical outcomes

We next conducted exploratory investigations on whether objective disease activity was concordant with clinical disease activity (Table 5). More than 50% of patients had evidence of discordance between objective disease activity and clinical remission at each core time point. Discordance rates in first trimester, second trimester, and third trimester were 83%, 56%, and 80%, respectively. Interestingly, only one patient with objective remission met the criteria for clinically active disease during this study. Of the 23 patients in clinical remission in second trimester, five also had evidence of objective disease activity (*p* = 0.002). Though not meeting significance, a similar trend was observed in third trimester, where 4 of 21 (19%) patients in clinical remission also met the criteria for objective disease (*p* = 0.059).

**Table 5:** Discordance between clinical disease activity and objective disease activity among pregnant patients with inflammatory bowel disease at core study time points.

Time Point		Objective remission	Objective disease	Total	*p* value
Trimester 1	Clinical remission	7	5	12	0.83
	Clinical disease	1	1	2	
	Total	8	6	14	
		Objective remission	Objective disease	Total	0.002
Trimester 2	Clinical remission	18	5	23	
	Clinical disease	0	4	4	
	Total	18	9	27	
		Objective remission	Objective disease	Total	0.06
Trimester 3	Clinical remission	17	4	21	
	Clinical disease	0	1	1	
	Total	17	5	22	

Clinical remission: mHBI < 5 or pMayo < 2; Clinical disease: mHBI ≥ 5 or pMayo ≥ 2.

Objective remission: FCP < 250 μg/g; Objective disease: ≥ 250 μg/g.

Bolded values represent discordance between clinical and objective assessment.

We also performed descriptive analyses between IBD disease activity and pregnancy-related outcomes in our cohort of patients. The median gestation at time of delivery in 28 pregnancies was 39.0 weeks (IQR 38.5–40.0). Of these, six (21%) patients had a spontaneous vaginal delivery, 11 (39%) had an induced vaginal delivery, 4 (14%) had a planned Cesarean birth, and 7 (25%) had an emergency C-section. Six of these seven (86%) emergency C-section deliveries had evidence of discordant disease activity during pregnancy, 3 (43%) in first trimester, 2 (29%) in second trimester, and 1 (14%) in third trimester. Twenty-seven (96%) deliveries were full-term (≥ 37 weeks), while only 1 (4%) was preterm (< 37 weeks). The median birth weight at delivery was 3276.3 g (IQR 3039.5–3496.3). Only one (4%) patient with discordant disease activity in first trimester delivered an infant preterm with LBW. Maternal IBD flares after delivery were documented in 5 (18%) patients; of these, three (60%) had evidence of active disease at baseline, one (20%) had an elevated FCP at third trimester, and one (20%) had clinical remission throughout the pregnancy.

## Discussion

Optimizing maternal and neonatal outcomes is essential to the care of pregnant people with IBD. In this pilot study, we demonstrate that remote monitoring of tight control management may be feasible and can be adopted in the care of pregnant people with IBD.

Our study results are promising as 77% of patients completed all core time points. However, we acknowledge that only 55% of patients with active disease completed the additional tight control monitoring time points. Reasons for not completing included pregnancy-related factors (i.e., time point coincided with delivery) or patients feeling anxious about a potential high FCP near delivery rather than feasibility of tight control management. This suggests that adherence is largely affected by patient-specific factors, which further highlights the importance of ongoing counselling and education in this subset of patients. Although our previous study demonstrated that a dedicated pregnancy clinic improves reproductive knowledge for people with IBD^35^, future work should aim to identify interventions that improve adherence with tight control management in this population. As such, our results suggest that tight control management via remote monitoring may have potential to complement routine clinical care in pregnant people with IBD.

We also demonstrate that remote monitoring is easy to implement during pregnancy for patients with IBD. The majority of patients expressed satisfaction using the IBDoc and IBD Dashboard and found both to be easy-to-use and user friendly. Although trialed in a limited cohort, all patients reported a strong preference for home point-of-care FCP monitoring over traditional approaches, suggesting a potential broad appeal of implementing these interventions in the management of IBD in pregnant people. These patients also strongly agreed that they would use the IBDoc in the future and would recommend it to other patients. These findings are consistent with previous studies enrolling non-pregnant patients with IBD^29–31^, which highlights that remote monitoring can supplement, and likely enhance, current management practices.

Incorporating eHealth and remote monitoring strategies are important in providing safe and effective means to monitor disease activity in pregnant patients with IBD. Relying on clinical scores or non-specific serum biomarkers of inflammation has led to variation and uncertainty in the management of IBD during pregnancy^36^. In this study, we demonstrate that more than 50% of patients had evidence of discordance between clinical and objective disease activity at each core time point. These results suggest that neither clinical symptoms nor objective markers of inflammation should be used in isolation to predict disease activity and escalation of therapy in pregnant patients with IBD. This is consistent with the CALM study^19^ where using both clinical symptoms and objective biomarkers of inflammation such as FCP to escalate therapy resulted in improved clinical and endoscopic outcomes.

Translating home point-of-care FCP monitoring into clinical practice may enable clinicians to improve care for pregnant patients with IBD. In particular, as the prevalence of IBD continues to rise, more people will carry the diagnosis during pregnancy, with an associated increase in related health care utilization and expenditure^6,37,38^. As such, the current strain and burden on existing health systems may serve as barriers in providing effective care to people with IBD during pregnancy^38,39^. Our results demonstrate that remote monitoring can overcome some of the challenges related to lab-based FCP testing, including time for analyses and patient convenience. Furthermore, it has been previously established that FCP is a surrogate marker for endoscopic disease activity and a strong predictor of long-term outcomes^10–18^. We have previously identified that elevated FCP may predict adverse pregnancy-related outcomes in patients with IBD^40^. As a result, it is possible that these interventions can be used for tight control management in order to reduce IBD-related adverse maternal and neonatal outcomes and related health care costs. We also demonstrate that no major safety signals were identified through home point-of-care FCP monitoring as maternal and neonatal outcomes were optimal. However, larger studies are necessary to evaluate the impact of tight control management on clinical outcomes for pregnant people with IBD.

The results of this feasibility study have identified measures to optimize the experience of eHealth programs and point-of-care testing for pregnant people with IBD. This will be critical in the implementation of our proposed large multi-center randomized controlled trial to assess whether tight control of IBD in pregnancy leads to improved maternal and neonatal outcomes. From our cohort of patients who were non-adherent to the additional monitoring time points, we identified that approximately half of these patients had previous pregnancy-related complications and associated antenatal anxiety. We also acknowledge that evidence of active disease during pregnancy can potentially further exacerbate anxiety in patients. In particular, we determined that 25% of non-adherent patients declined third trimester monitoring time points due to concerns of high FCP results near the time of delivery. Previous studies on non-pregnant patients with IBD demonstrated that anxiety and psychological distress decrease adherence to treatment^41^. While there was no assessment of mental health outcomes in our pilot study, incorporating self-reported screening questionnaires at each trimester into our future RCT may identify strategies for counselling and education to improve adherence. Furthermore, we identified that patients who were non-adherent to closer monitoring from active disease were predominantly from a higher socioeconomic group. Interestingly, a study led by Tomar et al. determined that higher education and upper socioeconomic status were negatively associated with adherence to treatment in patients with IBD^42^. However, our results suggest that adherence was largely driven by patient-specific factors rather than differences in socioeconomic status. Our efforts to improve adherence in future studies relies on adequate patient education prior to and during the study on the importance of FCP monitoring and point-of-care testing.

To our knowledge, this is the first study to demonstrate the feasibility of a tight control remote monitoring intervention for pregnant people with IBD. However, there remain inherent limitations of this study. In particular, a small sample size was recruited from a single clinic, which limit the external validity of the findings. Despite this, our findings suggest a need to conduct further investigations that assess the implementation of remote monitoring as a complement to standard clinical practice. Our future RCT study will be designed to compare clinical outcomes, service utilization, and costs of remote monitoring in pregnant people with IBD. In addition, we acknowledge that there is a limitation of recruiting patients from a single, academic center. Future studies that include pregnant people with IBD from different populations and health care settings are necessary to better evaluate the usability of remote monitoring interventions. Furthermore, despite the growing access to smartphones and internet technology, a population bias was inherent to our study design. Limited access to technology and wireless services will potentially limit the generalizability of our feasibility results for some patients.

## Conclusion

Increasing efforts to integrate eHealth programs in the management of chronic diseases has shifted the delivery of patient care beyond traditional clinical settings. Despite recent studies on the utility of remote monitoring in IBD, there has been no such trial in pregnant patients with IBD where optimization of disease activity is strongly recommended. This study has demonstrated that remote monitoring of tight control management is feasible among pregnant people with IBD and can be adopted into current clinical practice and health care settings. Integrating such solutions into routine care will enhance the delivery of real-time health data to both patients and health care providers. This has the potential to facilitate greater efficiency of care by allowing clinicians to implement a treat-to-target approach and provide tailored individualized care more readily.

Furthermore, the COVID-19 pandemic has stressed the need for safe and effective remote monitoring solutions following disruptions to traditional models of care. Although eHealth programs have been increasingly utilized due to in-person restrictions, challenges of virtualizing care for patients often exist. In particular, reduced availability of ambulatory clinics and increased patient aversion to clinical settings have highlighted the potential benefits of implementing home point-of-care monitoring for pregnant people with IBD. More importantly, exploring avenues to increase the utilization of remote monitoring in rural and remote areas can improve the quality of care for patients in these underserved communities. While remote monitoring is a promising solution, continued efforts are necessary to better evaluate its impact on patient care and management for pregnant people with IBD.

## Supplementary Information

Supplementary Information.

## Data Availability

The datasets generated during and/or analysed during the current study are available from the corresponding author on reasonable request.
