# Supplementary material for: Optimizing maternal and neonatal outcomes through tight control management of inflammatory bowel disease during pregnancy: a pilot feasibility study
Source: Sci Rep. 2023 May 22;13:8291. doi: 10.1038/s41598-023-35332-z (PMC10202356; doi:10.1038/s41598-023-35332-z)
Supplement: Supplementary file 1 — Supplementary Information. [file 41598_2023_35332_MOESM1_ESM.docx]

**Appendix 1**

| Rate the following statements from 1-5 |
| --- |
| (1 = strongly disagree, 5 = strongly agree) |
|  |
| **IBD Dashboard Questions** |
| 1.      The IBD Dashboard is easy to use |
| 2.      The IBD Dashboard is user friendly |
| 3.      The IBD Dashboard had too many questions |
| 4.      The questions on the IBD Dashboard took an appropriate amount of time to complete |
| 5.      I could easily report my clinical symptoms and medication intake on the IBD Dashboard |
| 6.      I could easily fit the time it took to complete the tasks on the IBD Dashboard into my schedule |
| 7.      The IBD Dashboard is helpful for tracking my symptoms |
| 8.      I feel comfortable providing my clinical symptoms and medication intake on the IBD Dashboard |
| 9.      The email reminders from the IBD Dashboard are useful |
| 10.  The IBD Dashboard is useful for monitoring disease |
| 11.  I liked using the IBD Dashboard |
| 12.  I would use the IBD Dashboard in the future |
| 13.  I would recommend the IBD Dashboard to other patients |
| **IBDoc Questions** |
| 1.      The IBDoc® tool kit is easy to use |
| 2.      The IBDoc® tool kit makes completing stool tests convenient |
| 3.      I could easily fit the time it took to complete the IBDoc® test into my schedule |
| 4.      The IBDoc® App is user friendly |
| 5.      I prefer completing at home stool tests using the IBDoc® tool kit (compared to taking samples to lab or clinic) |
| 6.      I would use the IBDoc® tool kit in the future |
| 7.      I would recommend the IBDoc® took kit to other patients |
